# Glandular trichome rupture in tomato plants is an ultra-fast and sensitive defense mechanism against insects

**DOI:** 10.1093/jxb/eraf257

**Published:** 2025-06-10

**Authors:** Jared Popowski, Lucas Warma, Alicia Abarca Cifuentes, Petra Bleeker, Maziyar Jalaal

**Affiliations:** Van der Waals-Zeeman Institute, Institute of Physics, University of Amsterdam, Amsterdam, The Netherlands; Van der Waals-Zeeman Institute, Institute of Physics, University of Amsterdam, Amsterdam, The Netherlands; Department of Plant Physiology, Green Life Sciences Research Theme, Swammerdam Institute for Life Sciences, University of Amsterdam, Amsterdam, The Netherlands; Department of Plant Physiology, Green Life Sciences Research Theme, Swammerdam Institute for Life Sciences, University of Amsterdam, Amsterdam, The Netherlands; Van der Waals-Zeeman Institute, Institute of Physics, University of Amsterdam, Amsterdam, The Netherlands; Technical University of Darmstadt, Germany

**Keywords:** plant biomechanics, plant–insect interactions, trichomes

## Abstract

Trichomes, specialized hair-like structures on the surfaces of many plants, play a crucial role in defense against herbivorous insects. We investigated the biomechanics of type VI glandular trichome rupture in cultivated tomato (*Solanum lycopersicum*) and a wild relative (*Solanum habrochaites*). Using micropipette force sensors and high-speed imaging, we uncovered the rupture mechanics underlying gland bursting, highlighting the small forces and short time scales involved in this process. Additionally, we observed larvae of the Western flower thrips (*Frankliniella occidentalis*), a major pest in tomato cultivation, inadvertently triggering trichome rupture and accumulating glandular secretions on their bodies. We developed a method to directly measure these insect-triggered rupture forces by analyzing the trichome stalk deflections during these interactions, which yielded forces of the same order of magnitude as our micropipette measurements. These findings demonstrate how rapid gland bursting and the fluid dynamics of glandular secretions act as an efficient and swift plant defense mechanism against insect herbivory.

## Teaser

Glandular trichomes function as ultra-fast biomechanical defense triggers in tomatoes, rupturing upon contact with insects and exposing them to sticky and toxic secretions.

## Introduction

Plants and the herbivores that consume them have been locked in an evolutionary arms race for hundreds of millions of years (Labandeira, 1997; Endara *et al*., 2017; Sharma *et al*., 2021). The fossil record shows evidence of plant tissue damage from insect predation essentially as far back as vascular plants have existed (Labandeira and Sepkoski, 1993; Boucot and Poinar, 2010; Pérez-de la Fuente *et al*., 2016). As a result, plants have developed increasingly more sophisticated defense systems against insects over time.

**Fig. 1.**
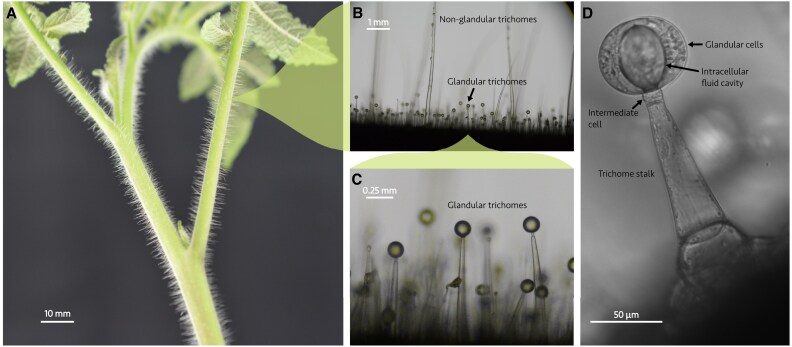
The multiscale nature of trichomes. (A) The stem and leaves of an *S. habrochaites* tomato plant, where the largest non-glandular trichomes are clearly visible as hair-like protrusions at distance from the plant surface. (B) A zoom-in on the stem of the same species, displaying a dense forest of different trichome types. The non-glandular trichomes are generally taller and extend out of the image frame, while the glandular trichomes have a typical height of ∼1 mm. (C) Close inspection of the short glandular trichomes. (D) A brightfield microscopy image (×20) of a type VI glandular trichome from an *S. habrochaites* leaflet sample, showing the fluid cavity stored within the head. The glandular head of each trichome is composed of four gland cells surrounding the intracellular fluid cavity, covered by a cuticle layer.

One such defense mechanism that has independently evolved numerous times and which is present in many plants (including most angiosperms) is trichomes (Wagner *et al*., 2004; Wang *et al*., 2021). Trichomes are small hair-like outgrowths present on the aerial tissues of a plant, consisting of differentiated collections of epidermal cells (Tissier, 2012; Chang *et al*., 2019). There is an enormous diversity of trichome types according to their shapes, number of constituent cells, and functions (Levin, 1973). Even for a given species, multiple types of trichomes can be present on the plant; species such as tomato can display up to seven different types (Luckwill, 1943). Trichome sizes range anywhere from a few micrometers to several centimeters (Tissier, 2012). However, a commonly applied classification distinguishes between glandular trichomes, which possess cells that produce and store specialized metabolites, and non-glandular trichomes, which lack these cellular factories (Fig. 1). Trichomes serve multiple purposes in plants, including water retention, temperature regulation, and reflecting light to reduce leaf absorptance (Wagner *et al*., 2004). However, defense against predation by insects is a primary role of trichomes, where they serve as mechanical and chemical barriers (Wagner, 1991; Shockley and Backus, 2002; Kennedy, 2003; Voigt *et al*., 2007; Peiffer *et al*., 2009). Strong correlations exist between trichome density and reduced insect reproduction, development rates, and oviposition, as well as increased mortality rates (Levin, 1973).

Current research on trichomes frequently focuses on the biochemical aspects of their defensive capacity, including pathways for specialized metabolite biosynthesis and mechanisms regulating their density and development (Peiffer *et al*., 2009; Bleeker *et al*., 2012; Bergau *et al*., 2015; Wang *et al*., 2021; Zabel *et al*., 2021; Han *et al*., 2022). These investigations attempt to exploit trichome defense compounds to enhance the resistance of a crop to pests through genetic approaches (Glas *et al*., 2012). Less attention has been given to the role of the physical properties of trichomes in plant defense (Bar and Shtein, 2019). Direct mechanical stimuli to trichomes are known to induce chemical signals originating at the basal cells of the trichome in both *Arabidopsis thaliana* trichomes and long digital (type I and II) trichomes of the cultivated tomato *Solanum lycopersicum.* This is evidence that trichomes in these species serve as sophisticated mechanosensitive switches that activate immune responses against predation (Zhou *et al*., 2017; Matsumura *et al*., 2022; Sun *et al*., 2024). Specialized hook trichomes can immobilize insects (Bustamante *et al*., 2017), while the rupture by insects of type VI trichomes in the *Solanaceae* family releases a toxic glandular fluid that impedes insect motion (Stringer, 1946; Gibson, 1971; Snyder and Carter, 1984). However, the physical mechanism by which glandular trichomes rupture under stress due to insect locomotion still remains elusive, particularly regarding the time scales and forces involved in this system.

In this work, we study the mechanical rupture of type VI glandular trichomes in two species of tomato, modern, cultivated tomato plants *Solanum lycopersicum* (cultivar) and the ‘hairy tomato’, a wild-type relative from the same genus, *Solanum habrochaites*, accession PI127826 (wild). The wild accession has a higher trichome density, different relative abundances of trichome types, and produces more glandular exudate in its trichomes than cultivar tomatoes (Snyder and Carter, 1985). Type VI trichomes are biosynthesis factories consisting of four glandular cells atop one intermediate cell and a single stalk cell (Tissier *et al*., 2017). We study type VI trichomes in particular because they are abundant on tomato stems and leaves, and they produce and store the vast majority of glandular fluid in this plant (Bergau *et al*., 2015). These trichomes do not passively secrete their glandular fluid, instead sequestering it in an intracellular fluid cavity in the space between the glandular cells (see Fig. 1D), from which it is released upon mechanical stimulation such as from insect locomotion. To simulate the effect of the impact of an insect’s leg in a reproducible fashion, we fabricated glass microcapillary tubes into precise force sensors (see the Materials and methods). We measured, for the first time, the force to rupture glandular trichomes and how rapidly the glandular fluid is released upon rupture. We then observed *in situ* insect-triggered glandular trichome rupture in experiments with the L2 larvae of Western flower thrips (*Frankliniella occidentalis*), demonstrating the effective defense strategy of the plant.

## Materials and methods

### Plant materials and growth conditions

Tomato plants from the wild accession PI127826 (*S. habrochaites*) and cultivar Moneymaker (*S. lycopersicum*) species were grown in a greenhouse facility that maintained consistent environmental conditions (24 °C; 60% humidity; 16/8 h light/dark cycle). Each plant was watered three times per week and fertilized twice per week on a regular schedule. Leaflets and stem tissues were harvested from plants of the same age, at the third leaf down from the shoot apex.

### Insect materials

Western flower thrips (*F. occidentallis*) were raised on cucumber plants in enclosed, temperature-controlled incubators under constant environmental conditions (22 °C; 48% humidity; 16/8 h light/dark cycle). For all experiments involving interactions with tomato, individual L2 larvae were collected in 1.5 ml Eppendorf tubes and transferred into a separate lab space for imaging, which was otherwise devoid of plants in order to avoid the spread of this invasive pest insect.

Insect experiments were performed by carefully transferring the thrips from the Eppendorf tubes into disposable pipette tips, the side of which was then gently tapped to place the insects onto the surface of the sample being imaged. Experiments were conducted on both stem and leaflet samples from each of the two tomato species studied here. Images and videos were then taken via a microscope of the thrips on the surface of the sample for up to 1 h after their deposition, before disposal of both the sample and thrips.

### Imaging

Bright-field microscopy was performed with a Nikon TI2 microscope using a halogen light source with a Photometrics BSI Express sCMOS camera. The glandular rupture force experiments involving micropipette force sensors utilized a ×4 objective lens with a numerical aperture of 0.13. High-speed imaging to better visualize the rapidity and localization of rupture was performed under the same microscope (×10 objective lens, numerical aperture 0.3) with a Phantom VEO 640L camera [28 000 frames s^–1^ (fps) sample rate], as well as with a horizontally mounted Nikon SMZ18 Stereo Microscope (×5 magnification) with a Chronos 2.1-HD High Speed Camera (2142 fps sample rate). The inner cavity morphology of type VI trichomes was imaged by mounting peripheral parts of leaflets on a microscope slide that is immersed in water, and imaging with a Thermo Fisher Scientific EVOSTM digital inverted microscope under bright light with a ×20 objective. Insect imaging was captured with both a Ximea MQ013MG-ON camera and a Nikon D5200 DSLR camera, attached to the horizontally mounted Nikon SMZ18 Stereo Microscope at various magnifications between ×1 and ×5.

### Micropipette force sensor manufacture and calibration

Micropipette manipulation provides opportunity to stimulate small-scale biological systems under microscopes and with high precision (Backholm and Bäumchen, 2019; Jalaal *et al*., 2020). To obtain our flexible micropipette force sensors, we started with a hollow glass capillary tube with 1 mm outer diameter and 0.75 mm inner diameter (TW100-4, World Precision Instruments, USA). A P-1000 micropipette puller system (Sutter Instrument, USA) with a pre-installed box filament (FB245B) was used to heat and rapidly pull this tube, which would split into two micropipettes with an ∼5 μm diameter tip at the end. The machine settings for micropipette pulling were consistent for all micropipettes manufactured (heat 750, pull 70, vel 150, time 200, pressure 100, ramp 709).

The end of the pulled micropipette was then cut and smoothed using a microforge (MF2, Narishige, Japan). This smoothing process (by holding the pipette end near a hot glass bead) prevented sharp tips, so that we could avoid piercing the trichome heads. With our experimental setup it was preferable to measure forces parallel to the end of the micropipette, so we created a right-angle bend near the tip of the pipette, also using the microforge. Hence, the straight portion of the pipette before the bend would act as the cantilever, and deflections for force measurements would be measured just before this bend. It was important for deflection analysis of the experimental data to be performed at approximately the same position along the pipette as in the calibration experiment because how much the pipette deflects under load depends on how far along it one measures.

Manufactured micropipettes were calibrated according to the water droplet and/or two-pipette deflection procedures described in Backholm and Bäumchen (2019). A custom ImageJ macro for extracting the pipette center of mass position and Python code used to analyze these calibration data according to the procedures used can be found at https://github.com/FluidLab/Micropipette-force-calibration. In experiments requiring micropipette translation, they were attached to SensaTex μMp-3 micromanipulators that have a high positional resolution. This enabled movement of the micropipette with a 5 nm resolution, and 100 nm repeatability.

Depending on where the pipette was cut and the location of the bend, the final tip diameter of the pipettes varied somewhat, with a range of 10–22 μm for the micropipettes used in our experiments. This led to a range of calibrated pipette *k*-values from *k* = 0.04 μN μm^–1^ to *k* = 1.17 μN μm^–1^, with typical uncertainties of the order ≤0.01 μN μm^–1^. For the force measurements using the deflection of these micropipettes, this corresponds to an uncertainty of tens to hundreds of nanonewtons, depending on the pipette and the resolution of the imaging camera.

## Results

### Mechanically weak cell junction leads to rapid rupture of glandular trichomes

Rupture of type VI glandular trichomes in wild and cultivar tomatoes was performed using micropipette force sensors under a bright-field microscope (see the Materials and methods). In practice, this consisted of loading either a stem or a leaflet sample under the microscope, bringing the trichomes at the edge of the sample into focus, and then moving the micropipette tip into frame using a micromanipulation stage. The tip would then be translated at a constant velocity towards the trichome glandular head until they made contact, where there would be a short period of force loading before the head ruptured.

**Fig. 2.**
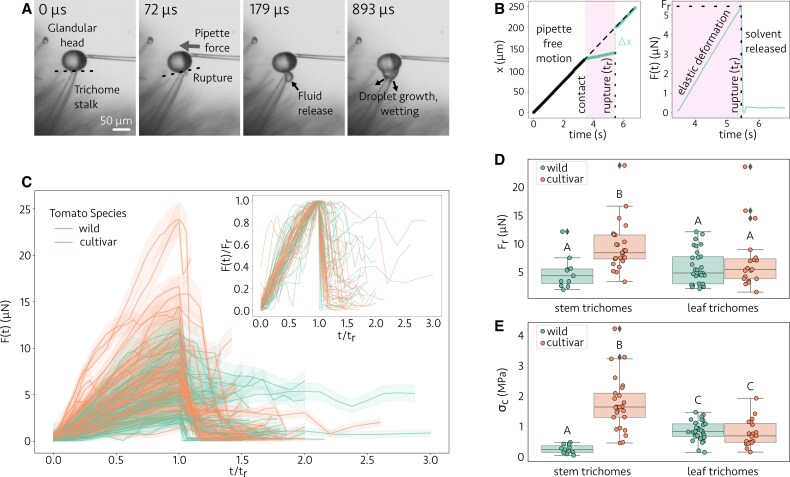
Mechanics of glandular trichome rupture. (A) Tomato trichome rupture occurs very rapidly upon the application of small amounts of force by a micropipette force sensor, with labeled times beginning from the last frame before rupture. The dashed line denotes the mechanically weak junction between the glandular and intermediate cells where rupture originates. These frames are from a high-speed video of type VI trichomes from a *S. habrochaites* tomato stem sample, filmed at 28 000 fps (Supplementary Video S1). (B) A representative experimental curve (different experiment from that in A) of the micropipette’s vertical center of mass location over time during trichome rupture (left), and the corresponding force loading curve (right). Two regimes are observed: pipette free motion before contact with the trichome head, and motion after the pipette makes contact (shaded region). Note that after rupture the curve returns to the same slope as the unobstructed curve. (C) Force loading curves for all measured ruptures of wild and cultivar tomato trichomes (*n* = 84). Shaded regions represent ±1σ (1SD) uncertainty bounds around the mean measured curves. The time axis for each curve is normalized by its measured time to rupture, so that each curve has its peak force (the force to rupture *F*_r_) at rescaled time *t*/*t*_r_ = 1. Inset: the loading curves collapse when both axes are normalized by their values at the point of rupture, demonstrating a universal fracture mechanism. Confidence intervals are removed for visual clarity. (D) The rupture forces for cultivar stem trichomes are significantly larger than for other species/trichome location pairs (largest *P*-value between pairs in Tukey’s honestly significant difference (HSD) test was *P* = 0.001). Diamonds denote outliers identified by the 1.5 interquartile range (IQR) method. (E) The critical stress to rupture trichomes is also larger for cultivar stem trichomes than for other species/location pairs with even higher significance, and the wild stem trichomes rupture at a significantly smaller critical stress than leaf trichomes. Above each box plot in (D) and (E), different letters indicate statistically significant differences between groups according to Tukey’s HSD test (*P <* 0.02), while groups sharing the same letter are not significantly different.

In all experimental trichome ruptures (*n* = 84), we consistently observed that rupture originated at the junction connecting the glandular trichome head and the intermediate cell of the trichome stalk. While we did not quantitatively measure the precise location of rupture in each experiment, its consistent localization was evident across all observations for both tomato species studied (see Supplementary Videos S1–S3, in which a pipette stimulates the glandular head from three distinct angles). This rupture occurs extremely rapidly, with the initial release of fluid occurring faster than the highest frame rate we could capture under the microscope with a ‘standard’ scientific camera (up to 500 fps). With the aid of high-speed imaging (up to 28 000 fps, see the Materials and methods), we discovered that the glandular fluid first emerges from its interior cavity on the order of 100 μs after the onset of detectable rupture, while the total time for its release is a factor of nearly 1000× longer and does not significantly differ between the two tomato species studied, with a combined mean value of 75 ± 35 ms (*n* = 28) (refer to Supplementary Appendix S1D for more details). The fluid reliably exits in the form of a droplet that wets the trichome stalk and grows in size until the fluid release halts. A time sequence of images capturing a single trichome rupture event is shown in Fig. 2A. There are no detectable jetting or spray phenomena, which indicates that surface tension spreading dominates the fluid dynamics of the problems. This can be quantified by estimating the non-dimensional numbers:


(1) $$Re=\rho \;U\;L/\mu ,\;We=\rho \;U^{2}\;L/\sigma ,\;Bo=\rho \;g\;L^{2}/\sigma ,\;$$

where ρ and σ are the density and surface tension of the liquid, respectively, estimated to be close to water, *g* is the gravitational acceleration, and *U* and *L* are characteristic velocity and length estimated from the observations. Note that neither surface tension nor density was measured directly (owing to the small sample volume and volatile nature of the fluids), so we have used the surface tension of water as an upper bound and the density of water as a lower bound. Nevertheless, our ensuing force estimates remain unchanged, since we do not expect substantial deviations in either property. The Reynolds number, *Re*, compares the inertial with viscous forces. The Weber number, *We*, measures the inertial to surface tension forces, and the Bond number, *Bo*, shows the ratio of gravitational to surface tension forces. We estimate the characteristic length as the typical size of the droplet released, that is about the radius of the intercellular cavity of the glandular head L∼O(10μm). From the high-speed images, the associated time scale to arrive at this length can be estimated as τ∼O(1ms), resulting in a characteristic velocity of $U\;∼O(10^{-2}\;m\;s^{-1})$. We measured the viscosity of the fluids in Supplementary Appendix S1B to be μ∼O(0.1−1Pa⋅s). Hence, the Reynolds number can be estimated as $Re∼O(10^{-4}-10^{-3})$, which is much smaller than unity, meaning that the viscous forces are much stronger than the inertial forces during the secretion of the fluid. Similarly, one can estimate the Weber and Bond number as $We∼O(10^{-5}-10^{-4})$ and $Bo∼O(10^{-5}-10^{-4})$, respectively, showing that the surface tension forces are also much stronger than inertial and gravitational forces. This places the secretion of glandular fluids at a low *Re*–*We*–*Bo* regime, which corresponds to a negligible effect of gravity and inertia and insufficient energy to produce jetting (Lohse, 2022; Challita *et al*., 2024). Instead, the fluid emerges as a droplet that rapidly spreads on the surface of the trichome stalk.

Note that the non-dimensional numbers are all based solely on observations of the fluid dynamics and not on the material properties and conditions leading to these dynamics, such as the internal pressure within the glandular head, the mechanical properties of the cells, or wetting properties of the glandular head and the stalk. During the rupture and ensuing first release of fluid, the radius of the glandular head did not noticeably decrease in size, which suggests that stored elastic energy is most likely to be negligible within this time frame. This contrasts with other rapid plant systems such as the seed-dispersal mechanism of *Ecballium elaterium* (the ‘squirting cucumber’), for which elastic energy storage is a key driver of the dynamics (Box *et al*., 2024).

Rupturing a trichome head with a well-calibrated micropipette force sensor (spring constant *k*) allows us to extract the applied force at all times from measurements of the deflection of the pipette Δ*x*. The pipette is translated at a constant speed ($\dot{\gamma }$ = 33 μm s^–1^ for all experiments) by a micromanipulator until contact with the trichome head, at which point the pipette slows down and increasingly deflects in the imaging plane until glandular rupture. For each time step during contact, we measure the center of mass location of the tip of the micropipette *x* to calculate Δ*x*, the relative difference in the trajectory of the pipette before versus during contact. The applied force is then


(2) F(t)=kΔx(t),

where *t* is time. A representative experimental curve for one trichome rupture of the pipette tip’s vertical center-of-mass location *x* and the corresponding force loading curve *F*(*t*) from Equation 2 are shown in Fig. 2B. The two constant-slope regimes of the pipette *x*-position during its free motion and after trichome contacts are indicative of its constant translation speed during these two intervals, with a shallower slope and thus slower speed after contact during force loading. After glandular rupture, the pipette returns to moving at the same speed as prior to the contact.

The collection of all force loading curves from the ruptures of both wild and cultivar tomato trichomes, sourced from the stems and leaves of the plants, is shown in Fig. 2C (*n* = 84). Despite rupturing after various times and at a range of forces (1–24 μN), the rupture processes were all quite similar. Specifically, the force curves consist of a period of elastic loading, indicated by a positive linear slope in Fig. 2C, followed by sudden failure, indicated by the steep drop in force to nearly zero due to trichome rupture removing contact between the glandular head and the pipette. Curves that do not drop entirely to zero force are the result of the glandular fluid sticking to the pipette head, providing a small resistive force to its motion. The consistency in rupture dynamics is demonstrated by rescaling the forces for each measurement by their peak force *F*_r_, shown in the inset of Fig. 2C. The observed scaling collapse indicates a universal fracture mechanism underlying the rupture, namely brittle fracture. Brittle fractures are characterized by a lack of substantial plastic deformation prior to failure and they propagate at extremely high speeds in a material, both of which are properties we observed in our rupture experiments (Field, 1971; Barsom and Rolfe, 1999; Divoux *et al*., 2024).

The maxima of each of the measured force loading curves define the rupture force *F*_r_. We compare the rupture forces between the two tomato species and the location of the trichome on the plant in the box plots of Fig. 2D. Aggregating all of the data for each species and location on the plant, we find a mean rupture force of *F*_r_ = 6 ± 3 μN [*n* = 79 after removal of five outliers using the 1.5 interquartile range (IQR) method]. However, the cultivar tomato stem trichomes had a significantly higher rupture force than other species/location pairs (*P* = 0.001 or smaller for all pairs following a two-way ANOVA test and post-hoc Tukey’s HSD test). Cultivar trichomes had rupture forces of *F*_r_ = 5 ± 2 μN (*n* = 16) from leaflet trichomes and *F*_r_ = 9 ± 3 μN (*n* = 24) from stem trichomes. Wild tomatoes had rupture forces of *F*_r_ = 6 ± 3 μN (*n* = 29) from leaflet trichomes and *F*_r_ = 4 ± 2 μN (*n* = 10) from stem trichomes.

Previous studies on type VI trichomes in tomatoes have observed a distinct boundary at the junction between the thick glandular cell wall and thinner intermediate cell wall (Bergau *et al*., 2015). We posit that external forces concentrate stress at this two-dimensional junction, triggering trichome rupture. To compute the stress concentration due to trichome bending, we model the trichome as an Euler–Bernoulli cantilever beam (Öchsner, 2021). Treating the trichome junction as a circular cross-section with measured radius *R*, its axial second moment of area is


(3) $$I_{z}=\frac{\pi R^{4}}{4}.\;$$

As the glandular head rotates at the moment of rupture (Fig. 2A), it is useful to quantify the torque experienced at the junction. Under the vectorial force *F* applied a distance vector *r* from the central axis of the junction, the trichome experiences a torque


(4) τ=r×F=|r||F|sinθ,

where θ is the angle between *F* and *r*. For each experimental rupture, we directly measured the rupture force, the distance *r* from the central axis of the junction to the point of force application on the glandular head, and the angle θ between these vectors. We then calculated the rupture torque using Equation 4 (see Supplementary Appendix S1A for results). Measurements of the torque to rupture showed similar results to that of *F*_r_, with a significantly higher torque needed to rupture cultivar stem trichomes than other species/location pairs. This indicates that our observed higher rupture force for cultivar stem trichomes is not explained by experimental variability in the pipette’s angle of force application, or by variations in the glandular head size among species/location pairs.

Under the applied torque τ from Equation 4, classical beam theory maintains that the junction’s stress field varies linearly with distance from its center and is maximal at the boundary of the interface (Öchsner, 2021). Denoting the torque to rupture by τ_r_, the critical stress on the boundary (distance *R* from the center) at the moment of trichome rupture is then


(5) $$\sigma_{c}=\frac{R\tau_{r}}{I_{z}}.\;$$

The radius of the intermediate cell *R* was measured and the critical stress calculated using Equation 5 for each of our experimental trichome ruptures (see Fig. 2E). The cultivar stem trichomes rupture at a critical stress that is significantly higher than other pairs. A new significant difference appeared for wild stem trichomes, which rupture at a smaller critical stress than the other pairs. These stress calculations, which account for the torque applied by the pipette regardless of angle, suggest that the rupture mechanism and differences between species are governed by intrinsic structural properties rather than just the magnitude of the applied force.

### Glandular trichome fluid acts as a mechanical barrier to insects

To validate the biological relevance of our laboratory measurements on the mechanics of tomato trichome rupture, we observe the *in situ* physical interactions of a pest with type VI trichomes on stem specimens from both tomato species. Insects are known to be impacted in various ways by the release of plant exudates, such as from glandular trichomes (Glas *et al*., 2012). In particular, adult Western flower thrips (*F. occidentalis*), a common pest of tomato plants, are known to have an avoidance response to the presence of volatile organic compounds produced by these glandular trichomes (Delphia *et al*., 2007). The larval stages of thrips lack wings and develop entirely on the leaves of tomato plants (Steenbergen *et al*., 2018). Hence, we chose to investigate trichome rupture dynamics with the L2-stage larva of the Western flower thrips (see the Materials and methods for details of their rearing).

**Fig. 3.**
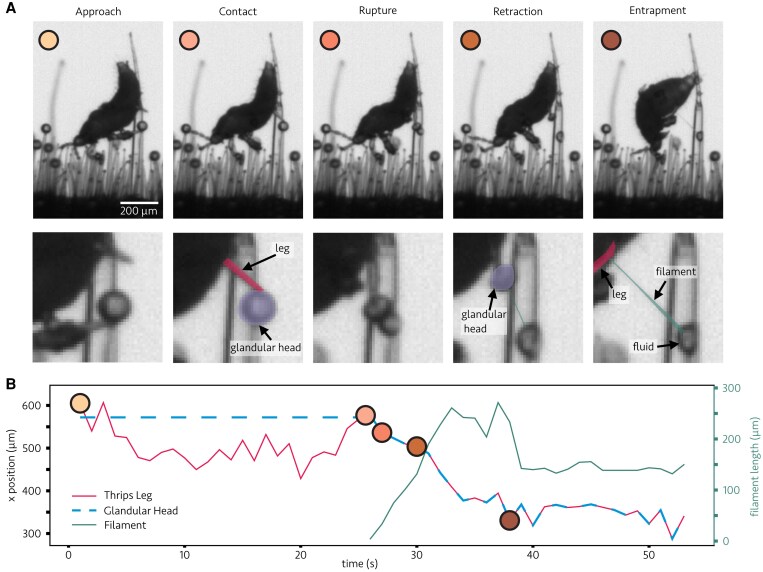
Thrips nymphs rupture tomato trichomes. (A) Frames from a microscopy video (Supplementary Video S4) before, during, and after a thrips leg causes type VI glandular trichome rupture on an *S. habrochaites* tomato stem sample. Zoomed-in panels are shown below each frame, with illustrative labels for the leg, glandular head, and fluid filament formed afterward. The colors assigned to the frames indicate the time of the experiment in the leg position versus time plot of (B) The plot also tracks the formation and length of the filament over the course of the video, as the insect struggles to move.

We observed numerous glandular trichome ruptures triggered by contact with the legs of thrips, with rupture occurring for both species of tomato (Supplementary Videos S4–S7). This rupture was occasionally followed by the formation of sticky filaments on the legs of the thrips, temporarily inhibiting their motion (see Supplementary Videos S4 and S5). A black-and-white bright-field microscopy video of a thrips on a section of wild tomato stem containing many type VI trichomes is presented as Supplementary Video S4. Several frames of this video, before, during, and after a thrips leg causes glandular trichome head rupture are displayed in Fig. 3A. We observed the glandular head becoming stuck to the leg, which then became held by a sticky filament secreted from the trichome after rupture. Over time, the thrips accumulate increasingly more fluid on their body. This filament appears viscoelastic based on our observation of rupture experiments with micropipettes. The chemical makeup of the fluid varies between species, but a major component for both species that contributes to the stickiness of their fluid is volatile terpenes (Xu *et al*., 2019; Kortbeek *et al*., 2023). These are stored in a lipid environment between the glandular cells, and are well known to have insecticidal effects and cause physical entrapment of the insects (Levin, 1973; Puterka *et al*., 2003; Simmons *et al*., 2004; Zabel *et al*., 2021). The fluid properties of these trichome fluids may function similarly to other plant-trapping fluids, such as the viscous digestive liquid in pitcher plant traps, where specific physical properties are essential for effectively capturing and retaining prey (Gaume and Forterre, 2007). We have estimated the viscosity of the trichome fluid using measurements of capillary flows inside the glass micropipette (see Supplementary Appendix S1B). Our preliminary results show that the viscosity of the liquid appears much larger than that of water (μ∼O(0.1−1Pa⋅s)), similar to that measured for the digestive liquids of pitcher plants. A detailed rheological characterization of these trichome secretions, using shear and extensional rheology, would help to reveal the physical mechanisms underlying their trapping success.

### Estimating insect-triggered rupture forces by using trichomes as force sensors

To further quantify the trichome–insect interactions observed in our experiments, we now extract the order of magnitude of insect leg forces causing rupture. Intuitively, if one has an estimate for the stiffness of a trichome stalk, then measurements of how much it deflects upon contact with a thrips leg can be used to extract the magnitude of the applied force of the insect. The stiffness of the trichome stalk is quantified by its geometry and its bending modulus *E*, measured in MPa. We performed independent measurements of the bending moduli for type VI trichome stalks, detailed in Supplementary Appendix S1C, by using our glass micropipettes to deflect these stalks and modeling them as homogeneous truncated cones. We find an average value of *E* = 55 ± 64 MPa (*n* = 19), where the large error bars on the standard deviation reflect a large spread in the measured values from *E* = 8 MPa to 235 MPa and the relatively small number of stalks sampled. Very few studies report trichome elastic moduli (Gorb *et al*., 2002; Bar and Shtein, 2019), and none provides bending moduli for tomato type VI trichomes against which our results can be compared.

**Fig. 4.**
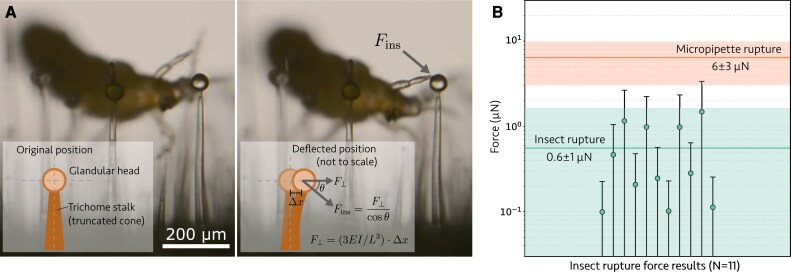
Type VI rupture forces under thrips nymph interactions, using trichomes as force sensors. (A) Upon contact with the trichome head, the trichome stalk deflects. We measured its deflection prior to rupture and the geometry of the trichomes, and applied our measurements for the bending modulus of type VI trichomes (Supplementary Appendix S1D) to extract the insect-triggered rupture force *F*_ins_. Frames are taken from Supplementary Video S6. Inset: schematics of the trichome’s initial and deflected positions, and the definitions of the forces measured. Here $I=\pi R_{1}^{3}R_{2}/4$ is the moment of area for a truncated cone cantilever, derived in Supplementary Appendix S1D. (B) The estimated insect-triggered rupture forces over *n* = 11 rupture events triggered by thrips in wild tomato (*S. habrochaites*) stem trichomes. Error bars on the data points extend to zero after the propagation of uncertainties in the bending modulus and trichome length measurements. Shaded regions show the mean with propagated uncertainty for the insect-triggered forces and the micropipette-derived rupture forces from Fig. 2C.

For each of the 11 insect-triggered ruptures that we considered, we measured the maximum deflection Δ*x* of the trichome stalk (measured just below the intermediate cell) under the applied force of a thrips leg immediately preceding the rupture (see inset of Fig. 4A). We also measured the stalk length of the trichome *L* up to the intermediate cell, the radii of the stalk just below the intermediate cell (*R*_1_) and at its base (*R*_2_), and the angle θ between the direction of the applied force and the axis of the trichome stalk. This was performed for all rupture events that we captured which had a discernible stalk deflection (*n* = 11, trichomes from wild tomato stem samples). To convert this deflection into a force, we invert Equation 15 for the bending modulus that was derived in Supplementary Appendix S1C,


(6) $$F_{⊥}=\frac{3\pi ER_{1}^{3}R_{2}}{4L^{3}}\cdot \Delta x,\;$$

and plug in our measured values for the trichome geometry and deflection. The symbol ‘ ⊥ ’ denotes that the force we extract from this deflection only measures the component of force that lies in the imaging plane, namely the component perpendicular to the axis of the trichome stalk. The full force applied by the insect is then given by


(7) $$F_{\text{ins}}=\frac{F_{⊥}}{\cos\;\theta }.\;$$

The results for the insect-triggered rupture forces are shown in Fig. 4B, compared with the micropipette-derived rupture forces of Fig. 2C. We find a mean value of *F*_ins_ = 0.6 ± 1 μN (*n* = 11), which is systematically lower than the micropipette results of *F*_r_ = 6 ± 3 μN (*n* = 79). We have higher confidence in the micropipette results due to the significantly larger number of statistics and precise calibration procedure. Additionally, the error bars for the insect rupture experiments extend down to zero due to the propagation of large uncertainties in the bending modulus results. The length of the trichome *L* was also not possible to accurately determine in many of the videos as its base was frequently not visible, and Equation 6 displays a strong sensitivity of the extracted force to this value of *L*. However, considering the large uncertainties in the insect-derived estimates, it is remarkable that they agree to within an order of magnitude with the micropipette-derived forces. This demonstrates a novel method for insect locomotion forces to be inferred, through observations of their interactions with well-calibrated mechanical barriers in their environment.

## Discussion

The consistency of the trichome rupture force loading curves in Fig. 2C is evidence of a universal fracture mechanical process underlying the dynamics in these species. This agrees with previous research that proposed the glandular head–intermediate cell junction as a ‘micro-abscission zone’ or breaking point for the release of the interior fluid, with rupture occurring under the application of ‘a light touch’ (Bergau *et al*., 2015). In this work, we provide direct experimental observations of this phenomenon, probing the full dynamics of the fluid release. Additionally, until now, the exact force needed to rupture the glandular head was unknown, apart from the assumption that small pest insects were capable of causing this rupture.

In our calculation of the critical stress, we modeled the type VI tomato trichome as a cantilever beam with a homogenous weak plane at the junction between the glandular cells and the intermediate cell. However, we acknowledge that our beam model represents a simplification of the true trichome structure. More accurately, the junction between the cell walls of the glandular head and intermediate cell surrounds an intracellular fluid cavity (Fig. 1D), and these thin cell walls probably bear most of the torque-induced stress. Lacking measurements of the cell wall thickness in this region, our model treats the cross-section as solid, potentially underestimating the actual stress experienced by the cell walls. Furthermore, variations in cell wall thickness between species and between stem and leaf trichomes are not accounted for in this analysis. Despite these limitations, the model provides a useful first-order approximation for comparing the relative stress applied to the junction of the trichome. A natural extension would be to conduct histological studies of glandular cell wall dimensions for the species/location pairs, alongside finite element method simulations in classical beam fracture mechanics, to refine our calculations and verify if our observed differences in the critical stress to rupture persist after accounting for structural variations (Anderson, 2017).

Morphologically, the concentration of stress at the glandular head–intermediate cell junction which leads to such consistent rupture dynamics may be a result of the developmental process of the storage cavity itself. Young glandular cells with cell walls that are initially in contact undergo localized lysis, gradually separating from the middle lamella and changing their chemical properties to make space for the storage cavity (Tissier *et al*., 2017). Pectin demethylation of the cell wall is known to be a critical part of cell wall separation in tomatoes (Bergau *et al*., 2015), and as the cavity expands to connect with the intermediate cell, it is possible that an overabundance of demethylated pectin becomes localized along the junction, providing a weak region for mechanical rupture. Additional study of the microscopic structures of trichome glandular heads could shed light on these predictions.

Within the tomato clade of the *Solanum* genus, previous research has established that type VI glandular trichomes defend against insect herbivory primarily through their production of high concentrations of toxic specialized metabolites contained in their glandular fluid (Tissier, 2012; Steenbergen *et al*., 2018; Kortbeek *et al*., 2019; Zabel *et al*., 2021). Our mechanical analysis now reveals the complementary physical characteristics of this defense system. We demonstrated that the ultra-sensitive rupture mechanics of type VI trichomes enables rapid fluid release upon insect contact, while the adhesive properties of the glandular fluid ensure that the toxic metabolites stick to insect appendages. In future work, long-term insect–plant interaction assays could be conducted, monitoring herbivore mobility and survival rate for various trichome densities as well as for mutants that are only missing the glandular heads for the type VI trichomes. The observation that L2 stage thrips larvae can rupture trichomes also provides a useful size threshold for understanding this defense mechanism. Previous studies have shown similar glandular rupture in the *Solanum* genus by larger insects such as aphids and cotton bollworms (Gibson, 1971; Simmons *et al*., 2004).

As shown in Table 1, many common tomato pests are equal to or larger in linear size than L2 thrips larvae (bold entries), suggesting that they could also trigger trichome rupture through normal movement. This indicates that trichomes may have evolved to rupture at forces corresponding to insects above a critical size threshold, effectively acting as a passive mechanical filter against herbivores. Note that the distribution of leg forces in insects may vary widely depending on the insect anatomy and environmental stresses (Full and Ahn, 1995; Delcomyn, 2004; Holmes *et al*., 2006), and furthermore locomotion forces do not scale linearly with insect mass, particularly for the smallest pests. Hence, linear size serves as a simple indicator for force distribution, and further research is needed to fully characterize the relationship between insect biomechanics and trichome rupture thresholds.

**Table 1.** Size comparison of common tomato pests, sorted by maximum body length*^a^*

Pest species	Life stage	Size (mm)	References
**Tobacco hornworm** (*Manduca sexta*)	Final instar larva	81.3	Madden and Chamberlin (1945)
**Cutworm** (*Agrotis ipsilon*)	Final instar larva	50.0	Capinera (2001)
**Brown stink bug** (*Euschistus servus*)	Adult	11.0–15.0	Capinera (2001)
**Tomato leafminer** (*Tuta absoluta*)	Adult	5.3–6.2	Bajracharya and Bhat (2018)
**Potato aphid** (*Macrosiphum euphorbiae*)	Adult	2.1–4.0	Capinera (2001)
**Green peach aphid** (*Myzus persicae*)	Adult	1.8–2.1	Capinera (2001)
**Greenhouse whitefly** (*Trialeurodes vaporariorum*)	Adult	1.0–2.0	Capinera (2001)
**Western flower thrips** (*Frankliniella occidentalis*)	Adult	1.2–1.9	Capinera (2001)
**Silverleaf whitefly** (*Bemisia tabaci*)	Adult	1.0–1.3	Capinera (2001)
Western flower thrips* (*Frankliniella occidentalis*)	L2 larva	0.7–0.8	Steenbergen *et al*. (2018)
**Greenhouse whitefly** (*Trialeurodes vaporariorum*)	Fourth instar larva	0.75	Capinera (2001)
Two-spotted spider mite (*Tetranychus urticae*)	Adult	0.4–0.5	Capinera (2001)
Silverleaf whitefly (*Bemisia tabaci*)	First instar larva	0.27	Capinera (2001)
Tomato eusset mite (*Aculops lycopersici*)	Adult	0.15–0.2	Capinera (2001)

^*a*^Bold formatting indicates pests of size equal to or larger than the L2 stage Western flower thrips (*), which were observed to rupture type VI glandular trichomes. This suggests that all bold-formatted pests are likely capable of rupturing these defensive structures through normal movement, while the non-bold pests may be unable to rupture them.

It is worth noting that our experimental setup for observing thrips–trichome interactions placed larvae directly on tomato stem sections with high trichome density. This scenario does not perfectly replicate natural conditions, as thrips typically develop on leaf surfaces where trichome density is lower and they can more easily navigate between trichomes. In natural settings, these insects probably seek paths that minimize trichome contact while moving across plant surfaces. Nevertheless, our observations provide valuable insights into what occurs during incidental trichome contact, which inevitably happens as insects navigate the plant.

In summary, we have measured the force to rupture the glandular heads of type VI tomato trichomes, in both wild-type and cultivar species. The rupture consistently originated at the junction between the glandular cells and the intermediate cell, in agreement with previous research on trichome development (Bergau *et al*., 2015). The rapidity with which the fluid contained in the glandular heads is released, occurring in <1 ms, adds trichome glandular rupture to a rare list of extremely rapid plant motions such as explosive seed and spore dispersal and elastic buckling mechanisms in some carnivorous plants, contrasting with the typically slow, imperceptible movements of plants (Vincent *et al*., 2011; Forterre, 2013; Box *et al*., 2024). The rupture force measurements had a mean value of 6 ± 3 μN (*n* = 79), with trichomes from the stems of cultivar tomatoes demonstrating significantly higher rupture forces than those from leaflets of the same plant or any location on the wild plants. The fluid makeup and volume in type VI trichomes are known to have slight biochemical variations across plant tissues, serving as one potential source of the significant differences between stem and leaf trichomes (Xu *et al*., 2019). Alternatively, differences in the cell wall junction thickness or makeup may concentrate different amounts of stress on the junction, affecting the force required for its rupture. A difference in cuticle composition between the species, in addition to their known differences in glandular cell morphologies, might also play a role. This provides the motivation for future biochemical studies. The increased mechanical resistance to rupture in cultivar stem trichomes probably compromises their defensive function, since effective pest deterrence relies on readily bursting trichomes that can quickly release their protective compounds upon insect contact. Historically, cultivated tomatoes have been selected mostly on valuable fruit characteristics such as fruit size, fruit consistency, and high yield. Perhaps this process may have inadvertently impacted the physical properties of the stem trichomes in particular.

Future research should examine additional wild tomato ancestors and characterize the molecular composition of the glandular–intermediate cell junction, to understand the genetic basis of trichome mechanical properties. Such insights into wild tomato defense mechanisms could guide breeding programs to recover robust pest resistance while maintaining the desirable traits of cultivated varieties, potentially reducing reliance on chemical pesticides. An immediate opportunity to build on this work is a more detailed rheological characterization of the gland-secreted fluids. Our observations indicate that, in addition to the well-known role of toxic chemicals within the fluid in repelling insects, the fluid also acts as a mechanical barrier by adhering to the animal’s legs and forming long filaments. Establishing a link between the glandular contents and the mechanical properties of the fluid (e.g. frequency-dependent viscoelasticity) could offer deeper insights into the evolution of plant defense mechanisms.

## Supplementary Material

eraf257_Supplementary_Data

## Data Availability

All data underlying this article are available at Zenodo (https://zenodo.org/records/15608443).

## Supplementary data

The following supplementary data are available at *JXB*  online.

Appendix S1. Supplementary results on the rupture torque (A), glandular fluid viscosity estimation (B), type VI trichome stalk bending moduli (C), and the total time for glandular fluid release (D).

Video S1. Micropipette-triggered rupture of a type VI wild tomato trichome, filmed at 28 000 fps.

Video S2. Micropipette-triggered rupture of a type VI cultivar tomato trichome, filmed at 28 000 fps.

Video S3. Total fluid release time for a micropipette-triggered wild-type VI trichome rupture, filmed at 2142 fps.

Video S4. Filament formation following wild tomato trichome rupture by a thrips nymph.

Video S5. Filament formation following cultivar tomato trichome rupture by a thrips nymph.

Video S6. Color video of a single trichome rupture event by a thrips nymph, on a section of wild tomato stem.

Video S7. Multiple trichome rupture events by a thrips nymph, on a section of wild tomato stem.

Video S8. Capillary flow of glandular fluid into a glass micropipette following rupture.
