# Supplementary material for: Glandular trichome rupture in tomato plants is an ultra-fast and sensitive defense mechanism against insects
Source: J Exp Bot. 2025 Jun 10;76(21):6508–19. doi: 10.1093/jxb/eraf257 (PMC12646162; doi:10.1093/jxb/eraf257)
Supplement: eraf257_Supplementary_Data [file eraf257_supplementary_data.zip › Appendix_S1.pdf]

APPENDIX S1

Supplemental Material: Glandular trichome rupture in tomato plants is an ultra-fast & sensitive defense mechanism against insects

Jared Popowski* and Lucas Warma

Van der Waals-Zeeman Institute, Institute of Physics,

University of Amsterdam, Science Park 904, Amsterdam, 1098XH, The Netherlands

Alicia Abarca Cifuentes and Petra Bleeker

Department of Plant Physiology, Green Life Sciences Research Theme,

Swammerdam Institute for Life Sciences, University of Amsterdam, 1098 XH

Amsterdam, The Netherlands

Maziyar Jalaal†

Van der Waals-Zeeman Institute, Institute of Physics,

University of Amsterdam, Science Park 904, Amsterdam, 1098XH, The Netherlands

* j.s.popowski@uva.nl

† m.jalaal@uva.nl

A. Rupture torque measurement

To ensure that our observed higher rupture force for cultivar stem trichomes is not due to the experimental variability in the pipette's angle of force application or the differing morphologies of glandular cells between species, we measured the torque to rupture (see Eq. 4 in the main text and the discussion about it). The results, shown in Fig. S1, are similar to that of the rupture force in Fig. 2D, with a significantly higher torque to rupture cultivar stem trichomes than other species/location pairs. Despite the two species having different glandular head morphologies, the physical size of the glandular head remains similar, such that observed variations in $\boldsymbol{r}$ arose more from differences in the pipette's location on the head than to the geometry of the head itself.

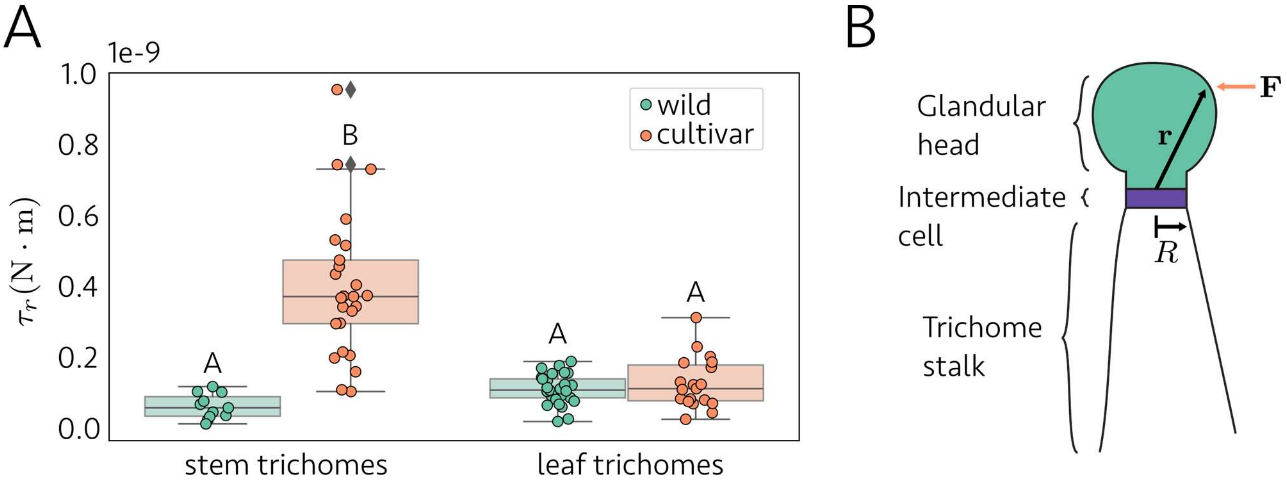

Fig. S1: **A.** Box plots of the torque to rupture tomato type VI glandular trichomes, for the two species and trichome locations on the plant studied in this work (N=84). Different letters indicate statistically significant differences between groups (p < 0.001), while groups sharing the same letter are not significantly different. **B.** Schematic of a trichome showing the definitions of **r**, **F**, and R used in Eqs. 4 and 5 of the main text.

B. Viscosity estimation

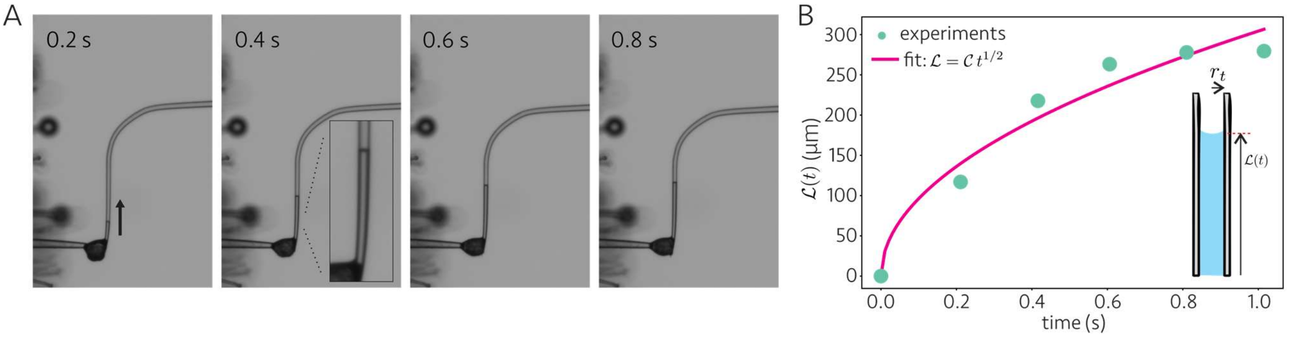

Fig. S2: **A.** Penetration of glandular fluid inside a glass micropipette by capillary flow over time. The direction of flow is indicated by an arrow in the first panel, and a magnified view is shown in the second panel. **B.** Variation of penetration length over time, where $\mathcal{C} = 304 \pm 13$ m/s$^{1/2}$ is the best-fit value of the coefficient in equation 6. Inset: a schematic highlighting the penetration length $L(t)$ and the radius of the tube, r_t.

Using a glass micropipette also enables us to (roughly) measure the fluid's viscosity (Fig. S2, Supplementary Video S8). When the glandular head bursts and the fluid is secreted, it rises through the pipette due to capillary action (Washburn, 1921; de Gennes et al., 2004). This dynamic process allows us to estimate the fluid's viscosity. We apply Washburn's equation, which describes the penetration length of a liquid in a wetting tube:

$$\mathcal{L}(t) = \mathcal{C}\, t^{1/2}; \quad \mathcal{C} = \sqrt{\frac{\sigma\, r_t \cos\theta}{2\,\mu}}. \tag{7}$$

Here, $\mathcal{L}$ and r_t represent the penetration length and the radius of the capillary tube, respectively (see the schematic in Figure S2B). The microcapillary tube gradually increases in radius from $r_1 = 4\mu\text{m}$ to $r_2 = 9\mu\text{m}$ over the range of $\mathcal{L}$ values that we plot here. We approximate this tapered cylinder as a cylinder of constant effective radius r_t. Since the volume of a tapered cylinder is

$$\frac{1}{3}\pi h(r_1^2 + r_1 r_2 + r_2^2), \tag{8}$$

for a cylinder to preserve this same volume it needs an effective radius r_t of

$$r_t = \sqrt{\frac{r_1^2 + r_1 r_2 + r_2^2}{3}}, \tag{9}$$

which for our system gives $r_t = 6.66\,\mu\text{m}$. Estimating the surface tension $\sigma = 72\text{mN/m}$ and the contact angle $\theta = 0^\circ$ as for water at room temperature, and our experimental best-fit value for the coefficient $\mathcal{C} = 304 \pm 13\ \text{m/s}^{1/2}$ in Eq. 7, we estimate the viscosity of the fluid to be $\mu = 2.6 \pm 0.1\ \text{Pa} \cdot \text{s}$. Given the lack of experimental repetitions and our uncertainties in the fluid's surface tension, contact angle, etc. we feel confident placing the viscosity in the range

$$\mu \sim \mathcal{O}(0.1 - 1\ \text{Pa} \cdot \text{s}).$$

This value falls within the range measured for certain plant digestive fluids (Gaume and Forterre, 2007) and corresponds to a fluid with significantly higher viscosity than water. We emphasize that our viscosity estimation using the present method suffers from various simplifications, including unknown surface properties of the liquid, as well as the neglect of other mechanical properties such as elasticity and extensional properties. In particular, the filament formation that we observed in Fig. 3 indicates that the glandular fluid is viscoelastic. Hence its viscosity is a function of the shear rate, and the shear rate will change as the fluid moves up through the capillary. Moreover, the fluid's highly volatile nature might rapidly influence this biological fluid's rheological properties, as we expect that viscosity rapidly increases as the volatile organics exit the solution and enter the surrounding air. These limitations highlight the need for future studies on the rheology of trichome glandular fluids.

C. Bending modulus measurement, truncated cone model

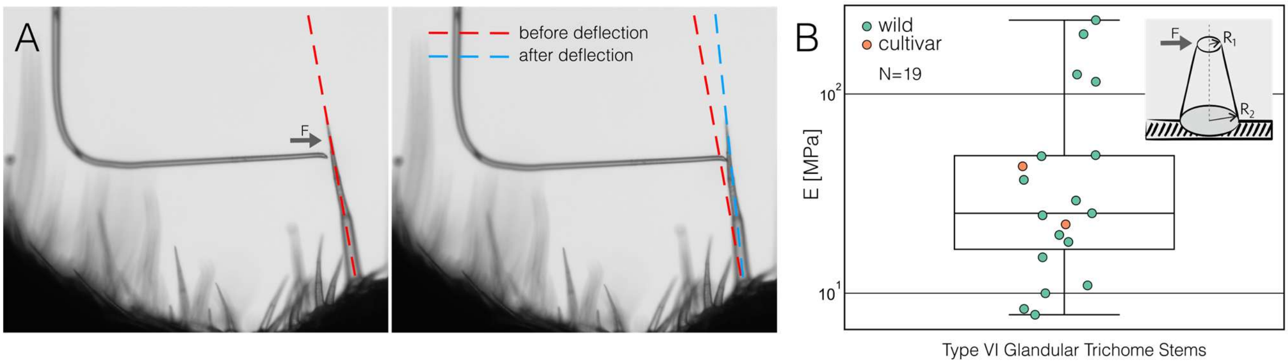

Fig. S3: **A.** Two frames from a trichome stalk deflection experiment, with the initial neutral axis (red dashed line) and deflected neutral axis (blue dashed line) labeled. Note that the tomato trichome shown here is type II and nonglandular, chosen because its large length makes the trichome deflection easier to see. For the type VI trichome experiments

reported here, stalk deflections were typically much smaller. **B.** The measured bending modulus values for type VI trichome stalks, ranging over more than an order of magnitude with a mean of $E = 55 \pm 64$ MPa. We emphasize the small number (N=19) of data points. Inset: the geometry of the trichome stalk, modeled as a solid truncated cone cantilever beam with an end-loaded force.

The bending modulus of type VI trichome stalks was measured by poking them with a micropipette, and measuring both the pipette's deflection and the stalk's deflection. The pipette's deflection tells us the force that we applied to the stalk, while the stalk's deflection under this applied force can be used to measure the bending modulus of the trichome, if one has a geometric model for the stalk and applies the equations of beam mechanics (Fig. S3).

Trichomes have a wide base where they emerge from the surrounding epithelial cells, with a gradual taper as one moves upwards towards the tip. We therefore model the trichome stalk as a truncated cone of height L with radii R_2 at the base and R_1 at the point of micropipette contact, just below the intermediate cell (Inset of Fig. S3B). The problem now takes the form of a truncated cone cantilever with an end-loaded force F. To solve for the cantilever's deflection, we apply the principle of virtual work, which says that the external work done by the pipette in deflecting the stalk is converted into internal (bending) energy,

$$W_{pipette} = U_{trichome} \,. \tag{10}$$

If the stalk deflects by a distance δ, the work done by the pipette is $W_{pipette} = \frac{1}{2}F\delta$, while the stored bending energy in the stalk is

$$U_{trichome} = \int_0^L \frac{M^2}{2EI}\,dx\,, \tag{11}$$

with bending moment $M(x) = F(L - x)$, bending modulus E, and second moment of area I. Generically, E and I are both functions of the distance x along the stalk, as the material composition and geometry may vary. For example, the cells that make up the tomato trichome stalk are known to contain non-uniform microtubule and actin filament networks, which gradually transition from transverse and spirally organized to longitudinal from the trichome's base to its tip (Chang et al., 2019). However, since we are concerned with how the trichome as a whole bends, we model the trichome stalk as consisting of a homogeneous material with a constant bending modulus E. At a fixed position x, the second moment of area is simply the same formula as in Eq. 3, $I = \pi R^4/4$, but now the radius R of the truncated cone's circular cross section is a function of x:

$$R(x) = R_1 - \left(\frac{R_1 - R_2}{L}\right)x. \tag{12}$$

Defining $b = R_1$ and $a = -\left(\frac{R_1 - R_2}{L}\right)$, the second moment of area as a function of x can be written

$$I = \frac{\pi}{4}(b + ax)^4. \tag{13}$$

Plugging this into Eq. 11 and applying the principle of virtual work (Eq. 10), the bending modulus of the trichome stalk satisfies

$$E = \frac{4F}{\pi\delta}\int_0^L \frac{(L - x)^2}{(b + ax)^4}\,dx\,. \tag{14}$$

This integral can be solved by the method of partial fractions, with the result

$$E = \frac{4FL^3}{3\pi\delta R_1^3 R_2}. \tag{15}$$

It should be noted that Eq. 15 reduces to the classic result for an end-loaded cylindrical beam when $R_1 = R_2$, as expected.

Thus, by measuring a given trichome's geometry (R_1, R_2, and length to applied load L) and the stalk's deflection δ, we can apply Eq. 15 to measure its bending modulus. This was done for 19 separate trials with type VI trichomes sourced from leaflet samples, primarily from the wild type tomatoes (Fig. S3B). We found a large spread in the measured values from $E = 8$ to 235 MPa, with a mean value of $E = 55 \pm 64$ MPa (N=19). These large error bars indicate that we have not taken enough data to converge on the population's true mean, and as such we treat these measurements as indicative of the order of magnitude of the bending modulus, for the purposes of calculating the forces applied by the thrips during trichome glandular rupture.

D. Total time for fluid release upon glandular rupture

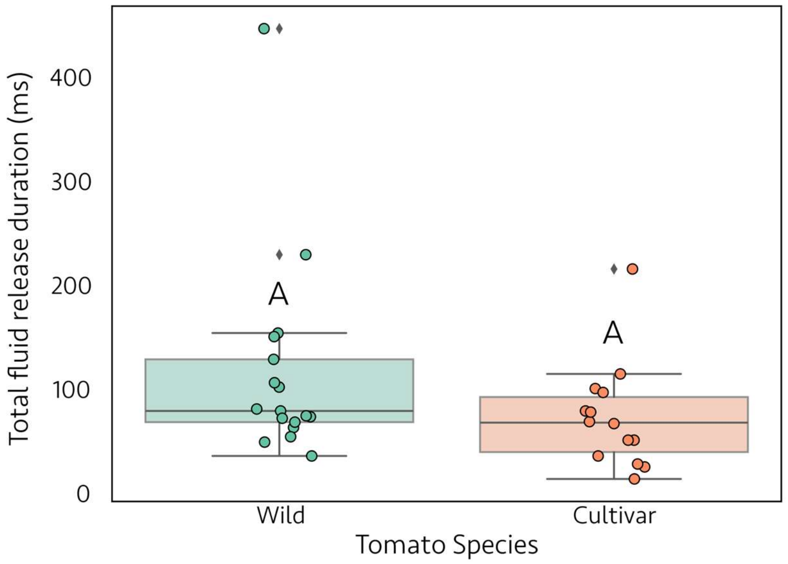

Fig. S4: **A.** The total fluid release duration for type VI trichome rupture from both tomato species studied. There is no statistically significant difference between the two species (p=0.18, Tukey's HSD test). We also found no statistically significant differences between the stem and leaf locations, or species/location pairs. The combined mean fluid release duration is 75 ± 35 ms (N=28).

We investigated the timescales of type VI trichome glandular rupture through controlled rupture experiments (N=31) with a micropipette while filming with a high-speed camera (see Materials and Methods for details). Videos were taken at both 2,142 fps and 28,000 fps. These experiments revealed that the time for the elastic rupture at the glandular head-intermediate cell junction to propagate from the rupture region until the first fluid was released is approximately 100 μs (see e.g., Figure 2A and Supplementary Videos S1 & S2), although the exact time was difficult to quantify as the initial fluid release was often the first detectable change in the glandular head following rupture. We anticipate that this timescale is related to the material properties of the glandular head-intermediate cell junction, which merit further study for its intriguingly sensitive biomechanical rupture capabilities.

Following the rapid rupture, we find that glandular fluid is released from the rupture site in the form of a droplet that grows as more fluid evacuates the cavity (see Supplementary Video S3). We quantified this timescale by the number of frames over which the droplet size grows after the rupture (notable by monitoring the dynamics of the glandular head). Fig. S4 plots our measurements of this fluid release timescale for both tomato species. We find that the droplet growth and wetting

of the trichome stalk happens rapidly (~100 ms), albeit nearly 1000x slower than the aforementioned elastic timescale. The wild tomato type exhibits a numerically longer fluid release duration (86 ± 35 ms, N=15 after removal of 2 outliers using the 1.5 IQR method) compared to the cultivated variety (63 ± 31 ms, N=13 after removal of 1 outlier), but Tukey's HSD test indicates that this difference is not statistically significant (p=0.18). There are also no significant differences between the location (stem/leaf), or between the species/location pairs. Thus, we combine all measurements and calculate a mean total fluid release duration of 75 ± 35 ms (N=28 after removal of 3 outliers) for type VI trichome rupture.

References

Chang J, Xu Z, Li M, Yang M, Qin H, Yang J, Wu S. 2019. Spatiotemporal cytoskeleton organizations determine morphogenesis of multicellular trichomes in tomato. *PLOS Genetics*, *15*(10), e1008438.

de Gennes P-G, Brochard-Wyart F, Quéré D. 2004. Capillarity and Wetting Phenomena: Drops, Bubbles, Pearls, Waves. In *Capillarity and Wetting Phenomena*. Springer New York.

Gaume L, Forterre Y. 2007. A viscoelastic deadly fluid in carnivorous pitcher plants. *PLoS ONE*, *2*(11), 1–7.

Washburn EW. 1921. The Dynamics of Capillary Flow. *Physical Review*, *17*(3), 273.
